# Corrupt practices negatively influenced food security and live expectancy in developing countries

**DOI:** 10.11604/pamj.2015.20.110.5311

**Published:** 2015-02-06

**Authors:** Florence Ngozi Uchendu, Thaddeus Olatunbosun Abolarin

**Affiliations:** 1 School of Health Sciences, National Open University of Nigeria, Victoria Island, Lagos, Nigeria; 2 Department of Mathematics and Statistics, Yaba College of Technology, Yaba, Lagos, Nigeria

**Keywords:** malnutrition, corruption, food security, live expectancy, population, developed and developing countries

## Abstract

Malnutrition is a global public health problem more prevalent in developing countries than in developed countries. Indicators of malnutrition include household food security and life expectancy. Corruption might be one of socio-political problems fuelling malnutrition in developing countries. The aim of this paper is to compare influence of corruption on food security, live expectancy (LE) and population in developed and developing countries. Thirty two least corrupt countries (LCC) and most corrupt countries (MCC) representing developed and developing countries were systematically selected using Corruption Perceptions Index (CPI). Countries’ data on population, food security index (FSI) and LE scores were obtained from Global food security index (GFSI) and Population reference bureau. T-test, Multivariate (Wilks’ Lambda), Pearson product moment analysis were performed to determine relationship between CPI, FSI, LE, and population in LCC and MCC at p<.05. Data were presented in tables, means and percentages. Mean CPI, Population, FSI, and LE in LCC and MCC were 71.5% and 24.2%; 34.8 and 41.7million; 75.0% and 37.4%; and 78.4years and 62.4years. There was a significant difference between CPI, FSI and LE in LCC and MCC (p < 0.05). CPI had a significant positive relationship with FSI and LE in LCC not MCC. There was also a significant relationship between FSI and LE in MCC. Low CPI influenced high FSI and LE in LCC while Low LE was associated with low FSI in MCC. Policies discouraging corrupt practices and promoting good governance should be embraced to eradicate malnutrition in developing countries.

## Introduction

Malnutrition is a global public health problem in developing countries especially Sub-Saharan Africa (SSA), India, Afghanistan, and South-Central/South-East Asia [1–3]. Globally, an estimated two billion people are affected by deficiencies of essential vitamins and minerals, collectively known as hidden hunger, which negatively impact on health and economic development [1]. One in three people in the world suffer from hidden hunger [2]. The recent Hidden hunger index reported that there were global hot spots of hidden hunger, with the prevalence alarmingly high in Sub-Saharan Africa as well as India and Afghanistan, and severe in many countries in South-Central/South-East Asia [2, 4]. Micronutrient deficiencies are silent epidemics of vitamin and mineral deficiencies affecting people of all genders and ages, as well as certain risk groups [5]. The most vulnerable are pre-school children, pregnant and lactating mothers and adolescents. In Nigeria, hidden hunger is a serious problem, with about 30% of children under-five estimated to be vitamin A deficient [6]. Studies have shown that over 34%-69% of childhood blindness in Nigeria is caused by corneal opacity, which results mainly from interplay of vitamin A deficiency, measles and harmful traditional eye practices [7]. About 75% are iron deficient and anaemic. Hidden hunger is a chronic lack of vitamins and minerals that often has no visible warning signs, so that people who suffer from it may not even be aware of it. It is as a result of inadequate intake of micronutrient dense staples and low food diversity. One carbohydrate staple can be eaten in different forms: swallowed, chewed, and drank, in a day as diet due to poverty. The affluent due to poor nutritional knowledge might eat more carbohydrate, saturated fat, high sugar and salty, ready-to-eat foods. These are found in some dining tables, fast foods, restaurants, eateries, supermarkets, and some airplanes. These foods are not cheap but are devoid of micronutrients and will only increase chances of obesity and cardiovascular diseases in individuals that over-indulge in them. Micronutrients are not generated by the body. They can only be obtained from daily food intakes in the right proportions. Such micronutrients include vitamin A, iron, iodine, zinc, and folate. If the diet of a particular individual is consistently devoid of these micronutrients, dietary deficiencies will be the consequence. Examples of micronutrient deficiencies are vitamin A deficiency (VAD), iodine deficiency disorder (IDD), iron deficiency and anemia (IDA), zinc deficiency, and folate deficiency. Animal and plant sources of micronutrients are meat, fish, liver, milk and milk products, green and yellow fruits and vegetables. Micronutrient deficiencies cause night blindness, poor physical growth, poor development, low intelligent quotient, low intellectual and cognitive abilities, neural bifida and thyroid dysfunction. Its consequences are disastrous: hidden hunger can lead to mental impairment, poor health and productivity, or even death.

Corruption is now the order of the day in public domain. It is very much part of the continuing debates on global public ethics and concerns about standards of behaviour in the government sector as well as in international business transactions [8]. Corruption thrives in societies where institutions of government are weak. Corruption widens the already yawning gap between the rich and the poor in many of the countries. It inhibits social and economic development, impacting negatively on attempts by international as well as regional development institutions to fight hunger and famine coherently and systematically. It distorts market operations. It deprives ordinary citizens of the benefits that should accrue to them, such as freedom from hunger in an age of plenty [8]. No initiative whether on food security or poverty alleviation or anything else for that matter will work in the absence of ethical public behaviour as a result of poor governance culture. Poverty alleviation and food security initiatives in Kashipur, India funded from 1977-1988 to the tune of 9 million British pounds from the International Fund for Agricultural Development failed as a result of systemic corruption [8]. Poverty is on the increase in Sub-Saharan Africa (SSA) and various forms of corruption threaten to undermine the impact of investments made to meet the Millennium Development Goals (MDGs) in the continent. The number of people who live on less than two dollars a day has doubled from 292 million in 1981 to nearly 555 million in 2005 [9]. Corruption and lack of government interest and investment are key players that must be addressed to solve the problem of malnutrition in Sub-Saharan Africa [10]. High levels of corruption stand at the epicenter of the food insecurity problems in Kenya. Corrupt governments cannot be expected to develop and implement sound long-term agricultural policies, including land tenure and water management, against a background of institutional instability [11]. Poor governance and corruption in major South Asian nations- India, Bangladesh and Nepal- is leading to widespread hunger in the regions. It have been indicated that the three countries have failed to ensure the citizen's right to food and record amongst the highest child malnutrition and maternal mortality rates in Asia [12]. Food security is the availability and accessibility of food nutritionally adequate in quantity, quality and variety at all times, to lead healthy and productive lives. Food insecurity is a chronic problem in many developing countries. The UN Office for the Coordination of Humanitarian Affairs (OCHA) recognizes five southern African countries with food security challenges, namely Angola, Madagascar, Malawi, Namibia and Zimbabwe. It has been estimated that 2.21 million people out of a total population of 13.1 million in Zimbabwe were affected by food insecurity: a 32% increase from the previous year. In Malawi, 1.5 million people were found to be food insecure and in Madagascar 3.9 million people [13]. Maplecroft data for food security risk index showed that in a survey of 197 countries, 59 are most at risk of food insecurity. Thirty-nine of these are African countries. Out of the 11 countries that are in the “extreme risk” category, nine are in Africa. They include Somalia and the Democratic Republic of Congo (DR Congo) (1^st^), Burundi (4^th^), Chad (5^th^), Ethiopia (6^th^), Eritrea (7^th^), South Sudan (9^th^), the Comoros (10^th^) and Sierra Leone (11^th^). Other countries that are at extreme risk category include Haiti (3^rd^) and Afghanistan (8^th^). The 48 countries considered to be at “high” risk for food supplies include Yemen (15^th^), Syria (16^th^), Niger (23^rd^), Pakistan (27^th^), Papua New Guinea (33^rd^), North Korea (35^th^), Mauritania (38), Mali (42^th^) and Burkina Faso (45^th^). High levels of governmental corruption and oppressive tactics against populations and political opposition are some of the factors responsible for this food insecurity crisis. When these factors combine with food insecurity, sparked by rising global prices, it can create an environment for social unrest and regime change [14, 15].

A study on what influence intelligent quotient (IQ) and/or corruption has on life expectancy found that corruption has a weak, but positive relationship (58%) on life expectancy, where 34% of the variation in life expectancy can be explained by corruption. When both IQ and corruption were combined, a strong positive relationship (80%), where 64 percent of the variation of life expectancy can be explained by the combination of corruption and IQ was observed. The study concluded that countries that are stupid and corrupt have a lower life expectancy, and vice versa [16]. The Nigerian Medical Association said that corruption among those in leadership position is responsible for poor infrastructure in hospitals, resulting in poor health indices and the consequent low life expectancy in the country [17]. In spite of the ills of corruption, and the numerous evidences to support them, there exist few studies that have compared the influence of corruption on food security, live expectancy and population in developed and developing countries to clearly pin-point the burden corruption places on developing countries. The purpose of this study is to fill this gap.

## Methods

### Sample collection

Countries were selected based on Corruption Perceptions Index (CPI) 2013. Out of 175 countries included in the 2013 CPI, 88 countries were selected as a representative sample. The 44 countries from LCC and MCC were selected each to represent developed and developing countries.

### Inclusion criteria

Countries were included as LCC if they have CPI > 50% and MCC if CPI was <50%. 32 MCC countries had complete data out of 44 selected and these were matched with 32 LCC countries.

### Data collection

Secondary data on countries’ corrupt practices, food security, population, and live expectancy scores were collected from published indexes [18–20]. Data from thirty two least corrupt countries (LCC) and most corrupt countries (MCC) were randomly selected respectively.

### Data analysis

Data were collected and arranged in a personal laptop in excel sheet. T-test, multivariate regression (Wilks’ Lambda) and Pearson product moment analyses were done to determine the relationship between corruption (CPI), food security (FSI), population and live expectancy (LE) in LCC and MCC at p<.05. Data were presented in tables, means and percentages.

### Ethics statement

The datasets used in this study were obtained from [18–20]. Full review of this study from an institutional review board was not sought as the datasets were anonymous and they are available for public use with no identifiable information on the survey participants.

## Results

Table 1 indicates the least corrupt and most corrupt countries (LCC and MCC) with their GFSI, population and LE figures. Mean CPI, GFSI, LE and population values for LCC and MCC were 71.5±13.0 and 24.2±4.7, 75.0±11.3 and 37.4±10.8%, 78.4±6.9 and 62.4±9.1 and 34.8±59.1 and 41.7±51.8. Table 2 shows the multivariate regression analysis of relationship between FSI, LE, population and CPI in LCC and MCC. Table 3 also shows a further comparism of the relationship between CPI, FSI, LE and population in LCC and MCC using pearson product moment correlation analysis.

**Table 1:** Mean CPI, FSI, LE and Population data of LCC and MCC

LCC		MCC									
S/N	Country	CPI** Score 2013/100	Pop.(≈ million)	FSI 2013	Live Expectancy (Both sexes)	S/N	Country	CPI** Score 2013/100	Population (≈ million)	FSI 2013	Live Expectancy (Both sexes)
1	Denmark	91	5.6	81.8	80	1	Sudan	11	34.2	25.2	62
2	New Zealand	91	4.5	82.0	81	2	Uzbekistan	17	30.2	40.9	68
3	Finland	89	5.4	81.4	81	3	Syria	17	21.9	36.7	75
4	Sweden	89	9.6	80.8	82	4	Yemen	18	25.2	29.6	62
5	Singapore	86	5.4	79.9	82	5	Haiti	19	10.4	27.6	62
6	Norway	86	5.1	86.5	81	6	Chad	19	12.2	22.1	50
7	Switzerland	85	8.1	83.2	83	7	Venezuela	20	29.7	60.8	75
8	Netherlands	83	16.8	83.2	81	8	Myanmar	21	53.3	40.1	65
9	Australia	85	23.1	80.1	82	9	Burundi	21	10.9	26.3	53
10	Canada	81	35.3	82.1	81	10	Tajikistan	22	8.1	34.2	67
11	Germany	78	80.6	81.7	80	11	Democratic Republic of Congo	22	71.1	20.8	49
12	United Kingdom	76	63.7	77.3	82	12	Angola	23	21.6	31.8	51
13	Belgium	75	11.2	82.4	80	13	Paraguay	24	6.8	52.9	72
14	Japan	74	127.3	77.8	83	14	Guinea	24	11.8	32	56
15	United States	73	316.2	86.8	79	15	Nigeria	25	173.6	33	52
16	Uruaguay	73	3.4	65.3	76	16	Cameroun	25	21.5	36.9	54
17	Ireland	72	4.6	81.7	81	17	Uganda	26	36.9	38.3	58
18	Chile	71	17.6	70.3	79	18	Kazakhstan	26	17	51.4	69
19	France	71	63.9	83.7	82	19	Honduras	26	8.6	48.4	73
20	Austria	69	8.5	83.4	81	20	Kenya	27	44.2	36.4	60
21	United Arab Emirate	69	9.3	65.7	76	21	Côte d′Ivoire	27	21.1	39.5	50
22	Botswana	64	1.9	60.0	47	22	Bangladesh	27	156.6	35.3	70
23	Portugal	62	10.5	76.1	80	23	Russia	28	143.5	60.9	70
24	Israel	61	8.1	78.4	82	24	Pakistan	28	190.7	39.7	66
25	Poland	60	38.5	69.9	77	25	Nicaragua	28	6	41.6	74
26	Spain	59	46.6	77.5	82	26	Mali	28	15.5	26.8	54
27	Korea (South)	55	50.2	71.1	81	27	Madagascar	28	22.5	29.3	60
28	Hungary	54	9.9	69.0	75	28	Togo	29	6.2	22.7	56
29	Costa Rica	53	4.7	63.7	79	29	Guatemala	29	15.4	45.2	71
30	Rwanda	53	11.1	29.3	63	30	Dominican Republic	29	10.3	51.9	73
31	Malaysia	50	29.8	64.5	75	31	Sierra-Leone	30	6.2	29	45
32	Turkey	50	76.1	62.9	74	32	Vietnam	31	89.7	48.6	73
Mean		*71.5±13.0	34.8±59.1	*75.0±11.3	*78.4 ±6.9	Mean		*24.2 ±4.7	41.7±51.8	*37.4±10.8	*62.3±9.1

** 100 = very good

* p < 0.05

**Table 2:** Multivariate analysis* of relationship between CPI, FSI, LE and population in LCC and MCC

	CPI			
	MCC		LCC	
	F-ratio	P-value	F-ratio	P-value
FSI	1.148	0.392	6.362	0.000**
LE	1.194	0.364	3.287	0.001**
Population	0.555	0.870	0.537	0.959

* Wilks’ Lambda

** Significant at p<.05

**Table 3:** Pearson product moment correlation analysis of relationship between CPI, FSI, LE and population in LCC and MCC

	MCC				LCC			
	CPI	Pop	FSI	LE	CPI	Pop	FSI	LE
CPI	1				1			
Population	0.196	1			−0.070	1		
FSI	0.284	0.147	1		0.650*	0.194	1	
LE	0.008	0.080	0.729*	1	0.395*	0.109	0.690*	1

* Significant at p<.05

## Discussion

### Corruption

The CPI index measures the perceived levels of public sector corruption in countries worldwide, scoring them from 0 (highly corrupt) to 100 (very clean). As the index decreased, corrupt practices and food insecurity increased. The MCC had a CPI which was three times lower than that of LCC and this is worrisome. The MCC are mainly from Sub-Saharan Africa (SSA) 46.9%, Asian countries 28.1%, Latin America and Caribbean 21.9% and Europe 3.1%. Developing countries in SSA and Asian countries constituted 75% of the MCC. Multivariate regression analysis of relationship between CPI, FSI, LE and population in LCC and MCC revealed that there was a significant relationship between low CPI and high FSI and LE in LCC while the reverse was the case in MCC. A positive relationship between corruption and food security as well as life expectancy implied that as corruption decreased food security and life expectancies increased. This result agrees with the findings of [16]. For LCC this might be due to availability of quality and up-to-date data, planned economy so that the effect of one can be easily seen in others. In LCC, economic resources are available for public welfare and this is why it has a positive influence on their GFSI (75.0±11.3) and LE (78.4±6.9) which are very high. This cannot be seen in MCC probably because there is no planned economy due to bad leadership, insecurity, frequent change of government and policies which makes it difficult to measure the impact of programs and projects. While the results of corruption are clear, the real extent of the problem is harder to pin down because corruption is shadowy and secretive by nature [18]. The public fund in MCC is diverted to personal use and so it cannot have any positive relationship with food security and live expectancy even though there could be other factors influencing them such as poverty, hunger, unemployment, low agricultural productivity, incessant war, and poor health facilities. This could be one of the reasons why GFSI (37.4±10.8) and LE (62.4±9.1) values are lower than that of LCC - GFSI (75.0±11.3) and LE (78.4±6.9). Corruption has a pernicious effect on food security [19]. Good governance, the antithesis of corruption, must be embraced and adopted wholeheartedly because it holds the key to food security on a sustained basis [8]. Developing countries must put in place stringent laws which must be implemented and executed without fear or favour to fight corruption. They should endeavour to have planned economy, quality and up-to-date data on their countries key indicators and continuous policies even when there is a change in government. The responsibility to deal with corruption is not just something for African governments and African people. They have a responsibility, I think, to help the poor countries in Africa recover some of their assets that were taken from them and deposited in banks in developed countries [21]. Corruption must be brought under control all over the world, as the poorer countries of the Third World especially cannot co-exist with it without being further dragged into the depths of untold human misery, starvation, disease, and degradation [8].

### Food Security

The gap between the GFSI of LCC and MCC was approximately 50%. Despite strong economic growth, food security remains an issue of primary importance for Africa. 75% of African countries have been at “high” or “extreme risk" [15]. Multivariate analysis showed that there was a significant relationship between corruption and GFSI of LCC (p<.05). High corruption in MCC might lead to poor welfare services and this might have a negative relationship with GFSI. Pearson product moment correlation analysis indicated that there was a strong positive relationship between GFSI and LE in MCC (Table 3). Poor food security in households could lead to malnutrition and low live expectancy in the populace especially among the poor.

### Life Expectancy

There was a significant difference in LE between LCC and MCC. The LCC had higher LE than the MCC. This might be as a result of low corrupt practices in these countries resulting in good economy and provision of basic amenities to the populace. Pearson product moment correlation analysis shows that there was a strong positive relationship between CPI, LE, and GFSI in LCC (p<.05). This result agrees with the findings of [16] and the opinions of [17]. Provision of basic amenities-adequate food, shelter, clothing and health care facilities-as a result of good governance and low corrupt practices positively influenced the GFSI and LE in LCC. As level of corrupt practices decreases in LCC, provision of subsidized adequate basic amenities is made possible and then life expectancy of the populace increases. Life expectancy was significant with food security in MCC indicating that poor food security definitely has a negative impact on life expectancy.

### Over Population

Many of the developing countries are over-populated even though there was no significant difference between the population in LCC (34.8±59.1) and MCC (41.7±51.7) (p>.05). CPI did not influence the population in both LCC and MCC even though it has been argued that population could influence corruption. Corruption also was not influenced by population. However, over population could lead to poor household food security, hunger and starvation, and malnutrition. The resultant effect could be low GFSI and LE in countries that do not have the resources to handle the situation.

## Conclusion

To achieve high GFSI and LE in the presence of high corruption in a country is a serious challenge in developing countries. This is evident from data in most corrupt countries (MCC) in this study. There was a relationship between minimal corruption practices and food security and life expectancy as seen in corruption “clean" countries (LCC). A multi-sectorial approach is crucial in tackling this problem. Improvement in government policies to discourage corrupt practices, promote good governance, primary health care, mechanized agriculture, household food security, availability of portable water, and nutrition education at community level should be embraced to eradicate malnutrition in developing countries.
